# Challenging the concept of microinvasive carcinoma of the vulva: report of a case with regional lymph node recurrence and review of the literature

**DOI:** 10.1186/1471-2407-6-157

**Published:** 2006-06-14

**Authors:** Jutta Sidor, Raihana Diallo-Danebrock, Elke Eltze, Ralph J Lellé

**Affiliations:** 1 Department of Obstetrics and Gynecology, St. Joseph's Hospital, Berlin, Germany; 2 Department of Pathology, Duesseldorf University, Germany; 3 Department of Pathology, Muenster University, Muenster, Germany; 4 Department of Obstetrics and Gynecology, Muenster University, Muenster, Germany

## Abstract

**Background:**

It is widely accepted that vulvar carcinoma with a depth of invasion of less than one millimeter is sufficiently treated by vulvectomy or wide local excision without inguinal lymphadenectomy.

**Case presentation:**

However, a patient with inguinal lymph node recurrence 21 months after radical vulvectomy for stage IA squamous cell carcinoma was observed.

**Conclusion:**

According to a review of the literature, there are five additional cases of metastasizing vulvar cancer with a depth of invasion of less than one millimeter. Therefore, the definition of microinvasive carcinoma of the vulva based on depth of invasion alone may not be as reliable as previously thought and does not rule out inguinal lymph node involvement or recurrence. Consequently, the necessity of inguinal node dissection for microinvasive carcinoma needs to be discussed on an individual basis taking into account the age of the patient as well as the potential morbidity of extended surgery.

## Background

Invasive vulvar carcinoma of all stages is usually treated by radical vulvectomy and bilateral lymphadenectomy. However, such an extensive surgical procedure has a high morbidity. In younger women, severe psychologic stress is caused by this hugely deforming operation, as sexual function and body image are disturbed. Therefore, over the past twenty years, efforts have been made to individualize treatment and to define a subgroup of patients which may be treated by less radical procedures [1]. It is widely thought that tumors with a depth of invasion of less than one millimeter according to criteria established by Wilkinson et al. [2,3] are sufficiently treated by vulvectomy or wide local excision without lymphadenectomy. However, there are five reports of lymph node metastases in patients with so-called "*microinvasive carcinoma of the vulva*". The patient reported here also developed inguinal lymph node metastasis after local treatment for squamous cell carcinoma of the vulva with a depth of invasion of less than one millimeter.

## Case presentation

**Figure 1:**
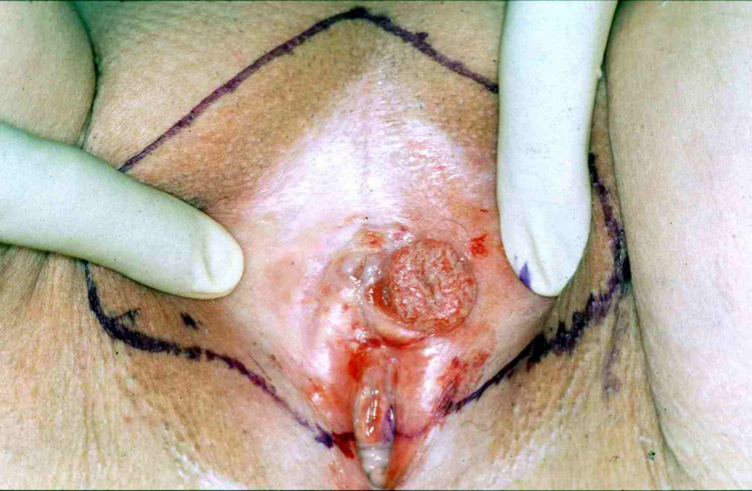
**Microinvasive squamous cell carcinoma of the vulva**. Initial presentation. Surgical resection margins are marked.

An 80-year-old white female was referred for evaluation of a suspicious vulvar lesion in September of 1997. At that time, the patient was free of complaints. On physical exam, atrophy of the vulva was seen but without evidence of lichen sclerosus. Approximately five millimeters from the clitoris, on the inner aspect of the left labium majus, a 1.5 cm indurated exophytic lesion was noted bleeding on touch (Figure 1). There was no involvement of the urethra or vagina. No regional lymphadenopathy was noted at this time. Biopsy revealed keratinizing squamous cell carcinoma. The patient had a previous history of recurrent adenocarcinoma within the oral cavity but had been free of disease for ten years.

**Figure 2:**
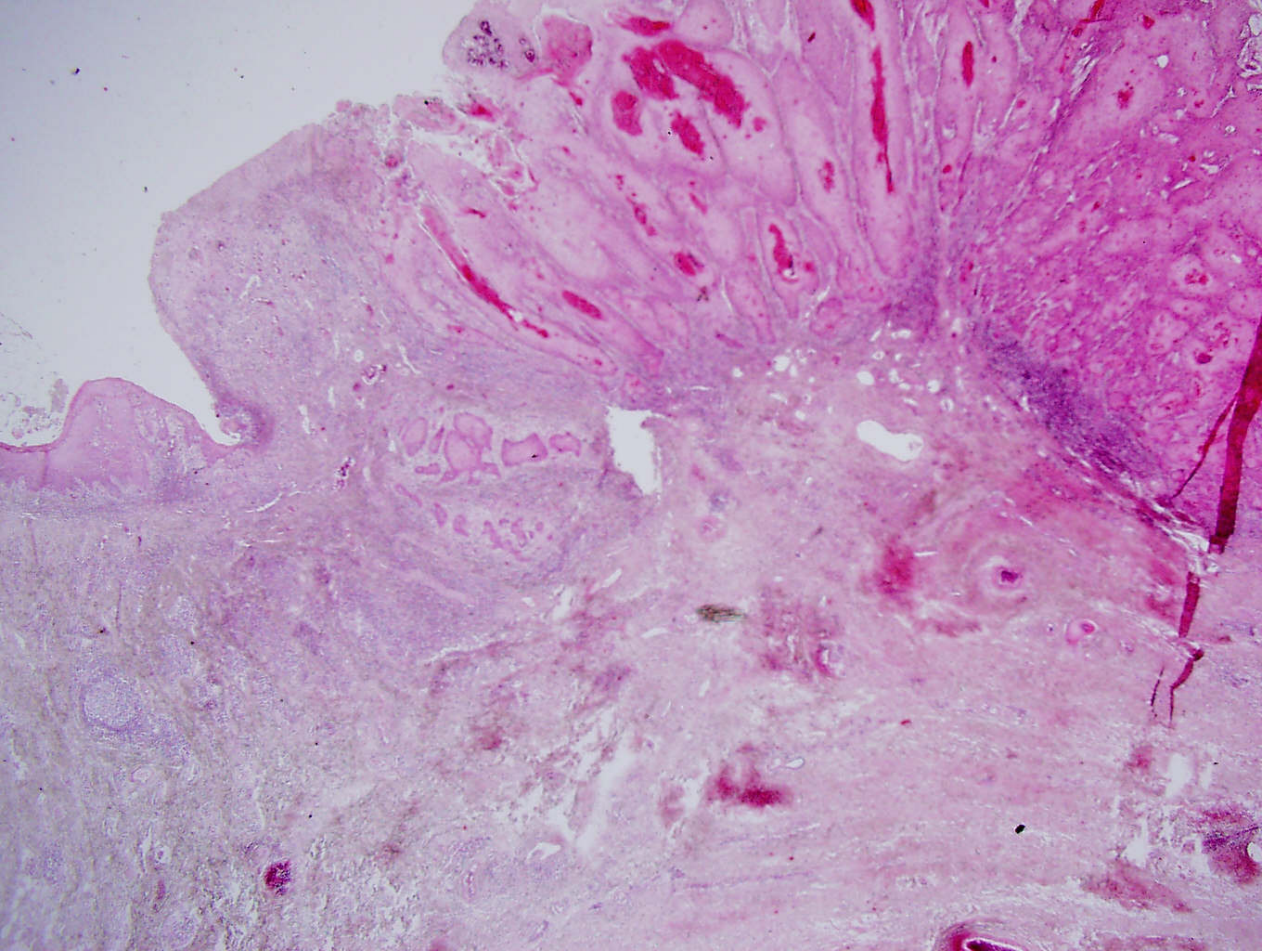
Photomicrograph of microinvasive squamous cell carcinoma of the vulva.

Anterior radical vulvectomy without inguinal lymph node dissection was performed with margins of at least 2 cm from the well defined lesion. Frozen section biopsy revealed a depth of invasion of less than 1 mm. Histopathologic evaluation of the entire specimen showed a well differentiated 1.7 × 1.5 cm superficially invasive keratinizing squamous cell carcinoma (Figure 2). The exophytic tumor had a thickness of 5 mm. The greatest depth of invasion was 0.08 mm using the criteria established by Wilkinson et al. [2]. No lymphvascular space involvement was seen and margins were tumor-free (at least 7 mm at the urethral margin as measured on the paraffin-embedded specimen).

**Figure 3:**
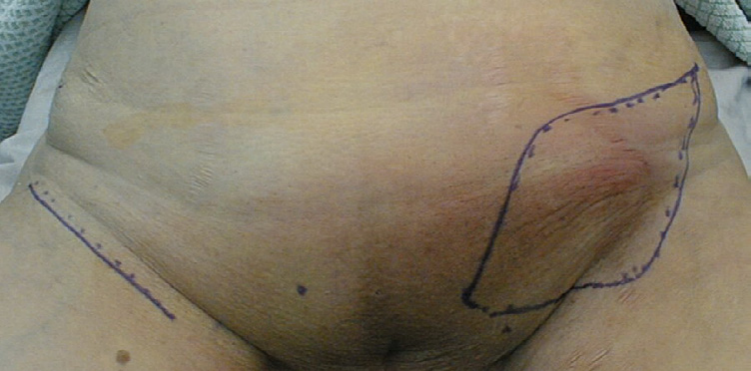
**Inguinal lymph node recurrence 21 months after treatment for microinvasive vulvar carcinoma**. Swelling of the left groin. Resection margins are outlined.

**Figure 4:**
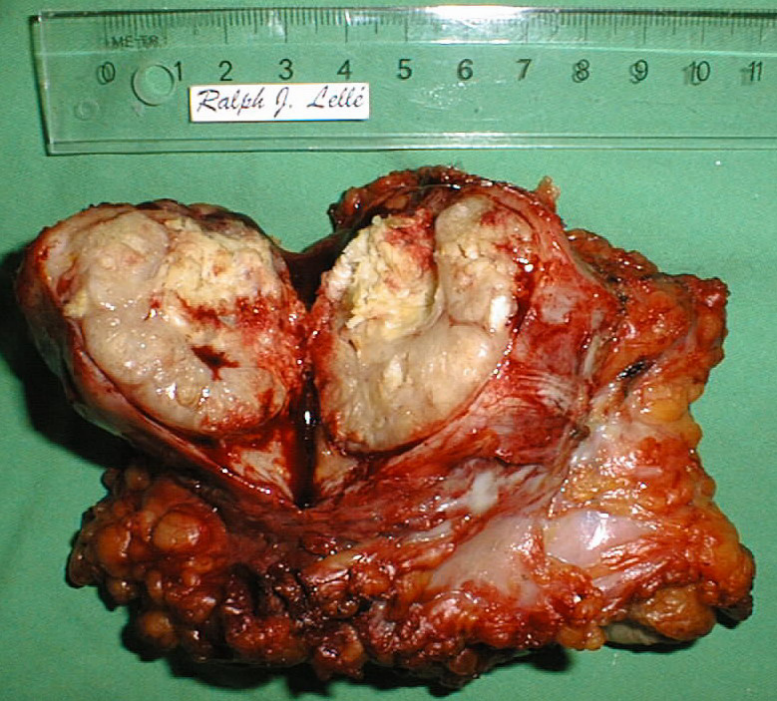
**Inguinal lymph node recurrence 21 months after treatment for microinvasive vulvar carcinoma**. The surgical specimen obtained from the left groin has been flipped over and incised.

The patient remained free of disease until twenty-one months later when she noticed a painful swelling of the left inguinal area (Figure 3). A five centimeter tumor was noted. A CT scan of the abdomen and pelvis did not show any enlarged pelvic or paraaortic lymph nodes. Subsequently, bilateral inguinal lymphadenectomy was performed. The left inguinal tumor was found to be a 4.3 cm metastasis of a well to moderately differentiated keratinizing squamous cell carcinoma consistent with the previous diagnosis of vulvar carcinoma (Figure 4). At that time, there was no evidence of vulvar recurrence and radiation therapy was given to the groin and to the pelvis. The patient remained free of disease for 69 months. She then developed extensive vulvar recurrence and was treated with palliative radiation therapy. She died 8 months later.

## Conclusion

So far, five cases of regional lymph node recurrences following treatment for FIGO stage Ia squamous cell carcinoma of the vulva have been reported in the literature [4-8]. One of two cases described by Thangavelu et al [5] cannot be considered true stage IA disease as the tumor was multifocal at initial presentation, the official FIGO classification stating that "Stage IA carcinoma of the vulva is defined as a single lesion measuring 2 cm or less in diameter with a depth of invasion of 1.0 mm or less ..." [9].

Age at initial diagnosis was between 39 and 84 years. Tumors were well differentiated (G1) in three patients [4,7,8] including the present case. None of the tumors showed lymphvascular space involvement. In four patients, vulvar intraepithelial neoplasia (VIN) was also present [4-6]. All patients with the exception of the case presented here had had a long-standing history of local complaints such as pruritus or suffered from vulvar diseases such as lichen sclerosus prior to the diagnosis of vulvar cancer.

The case reported by Volgger et al [4] is unusual as the patient had received immunosuppressive treatment for twelve years following a renal transplant. Long-standing Immunosuppression may have influenced the course of disease leading to groin node recurrence which occurred only four months after initial diagnosis, whereas, in the other five cases of microinvasive carcinoma, recurrence free survival was reported between 12 and 36 months.

All inguinal recurrences were unilateral. Five patients [4-6] including the present case died from progressive disease and/or distant metastases. In the other two cases [7,8], no follow-up information was provided.

In summary, no pattern of particular risk factors can be deducted from this small number of cases. Theoretically, HPV status of the tumor tissue may play a role. However, Pinto et al [10] did not find an association between HPV DNA in the primary tumor and clinical outome in patients with vulvar carcinoma, whereas viral presence in the inguinal lymph node metastases was indeed associated with a worse prognosis.

As the salvage rate for patients with inguinal recurrence is low, the development of groin metastases might have been avoided in some of these patients if inguinal lymphadenectomy had been performed at the time of initial cancer diagnosis [11]. In retrospect, the patient discussed in this article had been brought into remission through secondary lymph node dissection and radiation therapy. At the time of recurrence she was already 82 years old. In a younger patient, radiation therapy with the concurrent administration of cytotoxic treatment using cisplatin should have been considered.

A more extensive surgical approach including pelvic lymphadenectomy should be reserved for the debulking of grossly positive nodes. In all other cases the routine removal of pelvic lymph nodes is obsolete [12].

In conclusion, the definition of microinvasive carcinoma of the vulva based on depth of invasion alone is not as reliable as previously thought for ruling out inguinal lymph node involvement or recurrence. Although the present standard of care cannot be revised on the basis of a very small number of cases, the necessity of inguinal surgery for microinvasive carcinoma needs to be discussed on an individual basis taking into account the age of the patient as well as the potential morbidity. In addition, immunocompromised patients may be at a significantly higher risk for inguinal spread of vulvar cancer.

Thus, unilateral inguinal lymph node dissection through a separate incision should not be ruled out entirely in patients with microinvasive vulvar cancer. Certainly, radical groin node dissection with removal of the fascia lata and resection of the saphenous vein is not justified. Instead, the surgical procedure should be tailored to the exact localization of inguinal lymph nodes as defined by several anatomical studies [13-15].

Nevertheless, lymph node involvement is rare if depth of invasion is less than one millimeter, and omission of inguinal lymph node dissection may be discussed individually with selected patients in order to avoid the considerable morbidity following inguinal surgery. In the future, these patients should undergo sentinel lymph node biopsy. Although not yet applied routinely in vulvar cancer patients, preliminary studies show its feasibility: Sliutz et al [16] have been able to identify one or more sentinel lymph nodes in all 26 patients studied. Furthermore, the survival of patients with early vulvar cancer treated with sentinel lymph node biopsy and radical local excision appears to be excellent [17].

## Competing interests

The author(s) declare that they have no competing interests.

## Authors' contributions

JS performed the review of the literature and participated in the draft of the manuscript.

RD-D collected the specimen and performed the histopathological analysis.

EE participated in the histopathological analysis.

RJL took care of the patient and drafted the manuscript.

All authors have read and approved the final manuscript.

## Pre-publication history

The pre-publication history for this paper can be accessed here:
